# Supplementary material for: Multi‐dataset identification of innovative feature genes and molecular mechanisms in keratoconus
Source: J Cell Mol Med. 2024 Sep 19;28(18):e70079. doi: 10.1111/jcmm.70079 (PMC11412914; doi:10.1111/jcmm.70079)
Supplement: Supplementary file 2 — Material S2. [file JCMM-28-e70079-s002.zip › 7.GSEA analysis/3.DBNDD1/KEGG_GLYCOSAMINOGLYCAN_DEGRADATION.html]

Details for gene set KEGG\_GLYCOSAMINOGLYCAN\_DEGRADATION[GSEA]

|  || Dataset | DBNDD1.group.cls#h\_versus\_l |
| Phenotype | group.cls#h\_versus\_l |
| Upregulated in class | h |
| GeneSet | KEGG\_GLYCOSAMINOGLYCAN\_DEGRADATION |
| Enrichment Score (ES) | 0.31970152 |
| Normalized Enrichment Score (NES) | 1.0049806 |
| Nominal p-value | 0.4466403 |
| FDR q-value | 1.0 |
| FWER p-Value | 1.0 |
Table: GSEA Results Summary

  

Fig 1: Enrichment plot: KEGG\_GLYCOSAMINOGLYCAN\_DEGRADATION      
 Profile of the Running ES Score & Positions of GeneSet Members on the Rank Ordered List

  

| SYMBOL | TITLE | RANK IN GENE LIST | RANK METRIC SCORE | RUNNING ES | CORE ENRICHMENT || 1 | HS3ST3A1 | na | 876 | 0.396 | 0.2315 | Yes |
| 2 | ARSB | na | 7179 | 0.120 | 0.1280 | Yes |
| 3 | HPSE2 | na | 7392 | 0.116 | 0.1970 | Yes |
| 4 | HYAL1 | na | 7937 | 0.105 | 0.2493 | Yes |
| 5 | HYAL3 | na | 8741 | 0.090 | 0.2845 | Yes |
| 6 | GALNS | na | 10537 | 0.064 | 0.2743 | Yes |
| 7 | HYAL4 | na | 12057 | 0.046 | 0.2607 | Yes |
| 8 | NAGLU | na | 12663 | 0.041 | 0.2699 | Yes |
| 9 | HGSNAT | na | 12815 | 0.040 | 0.2913 | Yes |
| 10 | HEXA | na | 13363 | 0.035 | 0.2984 | Yes |
| 11 | SGSH | na | 14277 | 0.028 | 0.2903 | Yes |
| 12 | HS3ST3B1 | na | 14361 | 0.027 | 0.3056 | Yes |
| 13 | HYAL2 | na | 14473 | 0.027 | 0.3197 | Yes |
| 14 | HPSE | na | 17100 | 0.009 | 0.2497 | No |
| 15 | GNS | na | 17375 | 0.007 | 0.2464 | No |
| 16 | GLB1 | na | 18534 | -0.001 | 0.2137 | No |
| 17 | GUSB | na | 20109 | -0.014 | 0.1774 | No |
| 18 | IDUA | na | 21141 | -0.023 | 0.1628 | No |
| 19 | HEXB | na | 21627 | -0.028 | 0.1671 | No |
| 20 | IDS | na | 26923 | -0.106 | 0.0832 | No |
| 21 | SPAM1 | na | 30934 | -0.218 | 0.1093 | No |
Table: GSEA details [plain text format]

  

Fig 2: KEGG\_GLYCOSAMINOGLYCAN\_DEGRADATION      
 Blue-Pink O' Gram in the Space of the Analyzed GeneSet

  

Fig 3: KEGG\_GLYCOSAMINOGLYCAN\_DEGRADATION: Random ES distribution      
 Gene set null distribution of ES for **KEGG\_GLYCOSAMINOGLYCAN\_DEGRADATION**
